# Supplementary material for: Evaluating the Efficacy of Probiotics in IBS Treatment Using a Systematic Review of Clinical Trials and Multi-Criteria Decision Analysis
Source: Nutrients. 2022 Jun 28;14(13):2689. doi: 10.3390/nu14132689 (PMC9268703; doi:10.3390/nu14132689)
Supplement: Supplementary file 1 [file nutrients-14-02689-s001.zip › nutrients-1784226-Supplementary Table S1.pdf]

TITOLO ARTICOLO	AUTORE	NAZIONE PRIMO AUTORE	ANNO	GIORNALE	PRODOTTO	COMPOSIZIONE	TIPOLOGIA DI COMPOSIZIONE
of a multi-strain probiotic formulation (Bio-	Shamsuddin M. Ishaque,	Bangladesh	2018	BMC Gastroenterology	Probiotico	Bio-Kult	MIX PROBIOTICO
Reduces Depression Scores and Alters Brain	Maria Ines Pinto-Sanchez	Argentina	2017	Gastroenterology	Probiotico	Bifidobacterium longum NCC3001 (BL)	Bifidobacterium
Multispecies Probiotic Supplementation in	PhD	Italia	2019	Gastroenterology	Probiotico	CECT 4528 , Bifidobacterium animalis subsp. lactis Bi1 LMG P-17502,	MIX PROBIOTICO
improves equally with probiotic and placebo	Anna Lyra, Markku Hillilä	Finlandia	2016	Gastroenterology	Probiotico	Lactobacillus acidophilus NCFM	Lactobacillus acidophilus
Probiotics for Unconstipated Irritable Bowel	Joo Hyun Oh	Korea	2019	Nutrients	Probiotico	L. paracasei, L. salivarius, L. plantarum	MIX PROBIOTICO
symptoms and fecal microbiota in diarrhea-	Yi-Yuan Sun1,2, Ming Li1,	Cina	2018	Scientific Reports	Probiotico	Clostridium butyricum (CB)	Bacterium
bifidum MIMBb75 significantly alleviates	S. Guglielmetti	Italia	2011	and Therapeutics	Probiotico	Bifidobacterium bifidum MIMBb75	Bifidobacterium
treatments for irritable bowel syndrome in	Ahmet Baştürk	Turchia	2016	Gastroenterology	Simbiotico	Bifidobacterium lactis B94 with inulin	MIX SIMBIOTICO
Patients with Chronic Constipation: A	Jin Young Yoon,	Korea	2018	Sciences	Probiotico	plantarum LRCC5193	MIX PROBIOTICO
Controlled Trial: The Efficacy of Multispecies	Valerio Mezzasalma	Italia	2016	International	Probiotico	PBS067, Bifidobacterium animalis subsp. lactis BL050 (ex PBS075),	MIX PROBIOTICO
casei LBC80R and Lactobacillus	K. Preston	USA	2018	Beneficial Microbes	Probiotico	Lactobacillus rhamnosus CLR2	MIX PROBIOTICO
bowel syndrome: A randomized, double-blind,	Jun Sik Yoon	Korea	2013	and Hepatology	Probiotico	L. rhamnosus e Streptococcus thermophilus	MIX PROBIOTICO
assess the effect of a probiotic mixture on	K. Hod	Cina	2017	NGM	Probiotico	L. acidofilo; Bifidobacterium bifidum ; B. longum; B. breve; B. infanti;	MIX PROBIOTICO
probiotic vs. placebo in the irritable bowel	G. Sisson	UK	2014	and Therapeutics	Probiotico	NCIMB 30173, Lactobacillus acidophilus NCIMB 30175 e Enterococcus	MIX PROBIOTICO
Patients With Irritable Bowel Syndrome and A	Heidi MariaStaudacher	UK	2017	Gastroenterology	Simbiotico	Fod map/probiotico	MIX SIMBIOTICO
supplementation in the management of	Muhammed Majeed	India	2016	Nutrition Journal	Probiotico	Bacillus coagulans MTCC 5856	Bacillus coagulans
irritable bowel syndrome-related quality of	Vicente Lorenzo-Zúñig	Spagna	2017	Gastroenterology	Probiotico	Pediococcus acidilactici (CECT7483).	MIX PROBIOTICO
Inflammatory Biomarkers and Modifies the	Carmen Tenorio-Jiménez	Spagna	2019	Nutrients	Probiotico	Lactobacillus (L.) reuteri V3401	Lactobacillus reuteri
bifidum MIMBb75 (SYN-HI-001) in the	ViolaAndresenMD	Germania	2020	Gastroenterology &	Probiotico	Bifidobacterium bifidum MIMBb75	Bifidobacterium
Option for Patients with Irritable Bowel	Adrian Catinean	Romania	2019	Gastroenterology &	Simbiotico	Bifidobacterium longum W11e Bacillus spp.	MIX SIMBIOTICO
for probiotic (VSL#3) in irritable bowel	Reuben K Wong	Cina	2015	Dig Dis Sci	Probiotico	VSL#3: batteri lattici e bifidobatteri vivi e liofilizzati	MIX PROBIOTICO
with probiotic mixture on irritable bowel	Seok-Jae Ko	Korea	2011	Trials	Simbiotico	GJS/Doulac7S	MIX SIMBIOTICO
Antispasmodic Increases Quality of Life and	Diego A Barraza-Ortiz	Mexico	2020	Digestive Diseases	Probiotico	acidilactici CECT7483	MIX PROBIOTICO
on the Symptoms and Fecal Microbiota in	Ki Cha, Bong MD	Korea	2012	Gastroenterology	Probiotico	rhamnosus, Bifidobacterium breve, Bifidobacterium lactis,	MIX PROBIOTICO
and Bifidobacterium lactis UABla-12 Improve	Christopher J. Martoni	India	2020	Nutrients	Probiotico	lactis UABla-12)	MIX PROBIOTICO
and Bifidobacterium longum R0175 in	Erin D. lewis	Canada	2020	Nutrients	Probiotico	longum R0175	MIX PROBIOTICO
Abdominal Pain and Improves the Quality of	Giannetti E.	Italia	2017	Gastroenterology	Probiotico	Bifidobacterium infantis M-63	Bifidobacterium
Containing Lactobacillus and Bifidobacterium	Radomańska	Polonia	2020	Nutrients	Simbiotico	Fructooligosaccharides	MIX SIMBIOTICO
(DSM 9843) improves symptoms of irritable	Philippe Ducrotté	Francia	2012	Gastroenterology	Probiotico	Lactobacillus plantarum	Lactobacillus plantarum
animalis subsp. lactis, BB-12®, on defecation	Dorte Eskesen	Danimarca	2015	British Journal of Nutrition	Probiotico	Bifidobacterium animalis subsp. lactis, BB-12®	Bifidobacterium
probiotics and mosapride in patients with IBS	C. H. Choi	Korea	2015	Neurogastroenterology and	Probiotico	Bacillus subtilis and Strep tococcus faecium	MIX PROBIOTICO
Placebo-Controlled, Parallel-Group Study to	Tamar Ringel-Kulka	USA	2017	Gastroenterology	Probiotico	Bifidobacterium infantis 35624	Bifidobacterium
3856 as an add-on therapy for irritable bowel	Ravichandran Gayathri2	India	2019	Colorectal Disease	Probiotico	Saccharomyces cerevisiae CNCM I-3856	Saccharomyces
effect of a multispecies probiotic on	S Ludidi	Netherlands	2014	Neurogastroenterology and	Probiotico	salivarius W57, Lactococcus lactis W58, Lactobacillus acidophilus	MIX PROBIOTICO
probiotic Bacillus coagulans Unique IS2 vs.	Ratna Sudha Madempudi	India	2019	Scientific Reports	Probiotico	Bacillus coagulans Unique IS2	Bacillus coagulans
irritable bowel syndrome: An individual	Amélie Cayzeele-Decherf	Francia	2017	Gastroenterology	Probiotico	Saccharomyces cerevisiae (S. cerevisiae) CNCM I-3856	Saccharomyces
1917 in Patients with Irritable Bowel	Amir H. Faghih	Iran	2015	Internal Medicine	Probiotico	E. coliNissle 1917	Bacterium
freeze-dried culture in irritable bowel	Michel Dapoigny	Francia	2012	Gastroenterology	Probiotico	Lactobacillus casei	Lactobacillus casei
controlled Multicenter Trial of Saccharomyces	Choi, Chang Hwan MD	Korea	2011	Gastroenterology	Probiotico	saccharomyces boulardii	Saccharomyces
Lactobacillus gasseri strain CP2305 on quality	K Nobutani	Giappone	2017	J Appl Microbiol	Probiotico	Lactobacillus Gasseri strain CP2305	Lactobacillus gasseri
of Lactobacillus plantarum 299 v on symptoms	CherylStevensonM.Sc.	Lexington County	2014	Nutrition	Probiotico	Lactobacillus plantarum	Lactobacillus plantarum
multi-strain probiotic in Iranian adults with	Elham Jafari	Iran	2014	Archives of Iranian Medicine	Probiotico	12®, Lactobacillus acidophilus LA-5®, Lactobacillus del-brueckii subsp.	MIX PROBIOTICO
primary care patients with irritable bowel	Luise Mølenberg Begtrup	Danimarca	2013	Gastroenterology	Probiotico	and Bifidobacterium Bb12	MIX PROBIOTICO
'functional food' in the management of	Lesley M Roberts	UK	2013	BMC Gastroenterology	Simbiotico	number I-1630) and L. bulgaricus (CNCM strain numbersI-1632 and I-	MIX SIMBIOTICO
symptoms and intestinal flora in patients with	Bo Søndergaard 1	Danimarca	2011	Gastroenterology	Probiotico	and Bifidobacterium lactis Bb12	MIX PROBIOTICO
patients with irritable bowel syndrome	Annemieke Y Thijssen	Netherlands	2015	Hepatology	Probiotico	Lactobacillus casei Shirota	Lactobacillus casei
Lactobacillus acidophilus La-5 and	Bojana Bogovič Matijašić	Slovenia	2016	Journal of Dairy Science	Simbiotico	and Bifidobacterium animalis ssp. lactis BB-12	MIX SIMBIOTICO
controlled clinical trial on efficacy and safety	R Urgesi	Italia	2014	Medical and	Simbiotico	Simethicone / Colinox	MIX SIMBIOTICO

mixture in patients with irritable bowel	Carmelina Cappello	Italia	2013	Int J Colorectal Dis	Simbiotico	lyophilised bacteria (5×109 Lactobacillus plantarum, 2×109	MIX SIMBIOTICO
Saccharomyces boulardii therapy in diarrhea-	Zaigham Abbas	Pakistan	2014	Eur J Gastroenterol Hepatol	Probiotico	Saccharomyces boulardii	Saccharomyces
acidophilus and bulgaricus) utility in the	Javier Diaz Ferrer	Perù	2012	Rev Gastroenterol Peru	Probiotico	Lactobacillus acidophilus	Lactobacillus acidophilus
35624 and changes in fecal microbiota after	Duane Charbonneau	Irlanda	2013	Gut Microbes	Probiotico	Bifidobacterium infantis 35624	Bifidobacterium
treatment of irritable bowel syndrome in	M Ratna Sudha 1	India	2018	Beneficial Microbes	Probiotico	Bacillus coagulans Unique IS2	Bacillus coagulans
Lactobacillus brevis KB290 and β-carotene on	Nobuo Fuke	Giappone	2017	Food Sciences and Nutrition	Simbiotico	Lactobacillus brevis KB290 and β-carotene	MIX SIMBIOTICO
acacia fiber and Bifidobacterium lactis	Yang Won Min	Korea	2012	Gastroenterology	Simbiotico	Yogurt enriched with acacia fiber and Bifidobacterium lactis	MIX SIMBIOTICO
Probiotic Preparation in Patients with	Radomańska	Polonia	2021	Nutrients	Probiotico	Lactobacillus, Bifidobacterium Streptococcus thermophilus	MIX PROBIOTICO
predominant irritable bowel syndrome	M A Kabir	Bangladesh	2011	Journa	Probiotico	Saccharomyces boulardii	Saccharomyces
mixture in irritable bowel syndrome: an open-	C Bucci	Italia	2013	Tech Coloproctol	Simbiotico	Lactobacillus gasseri, Bifidobacterium infantis e Bifidobacterium	MIX SIMBIOTICO
rhamnosus GG in irritable bowel syndrome	Natalia Pedersen	Danimarca	2014	Gastroenterology	Simbiotico	(LFD) and the probiotic Lactobacillus rham nosus GG (LGG)	MIX SIMBIOTICO
symptoms in IBS patients independent of	Elsa Sandberg Nielsen	Danimarca	2018	Food Funct	Prebiotico	Lacto-fermented sauerkraut	Prebiotico
predominant irritable bowel syndrome: A	Anca Trifan	Romania	2019	United European	Prebiotico	Gelsectan: XG, PPT e XOS	Prebiotico
Hydrolyzed Guar Gum on Fecal Characteristics	Zenta Yasukawa	Giappone	2019	Nutrients	Prebiotico	PHGG	Prebiotico
Low FODMAP Diet Improves Irritable Bowel	Bridgette Wilson	UK	2020	Gastroenterology	Prebiotico	β-galactooligosaccharides (B-GOS)	Prebiotico
alter the colonic luminal microenvironment	Emma P Halmos	Australia	2015	Gut microbiota	Prebiotico	Foodmap	Prebiotico
luminal bifidobacteria and gastrointestinal	Heidi M Staudacher	UK	2012	Nutrition and Disease	Prebiotico	Foodmap	Prebiotico
predominant irritable bowel syndrome	Lin Xu	Cina	2015	Ke Za Zhi	Prebiotico	Pectina	Prebiotico
microbiota composition in a clinical trial of	K. Hod	Israele	2018	Neurogastroenterology and	Probiotico	L. acidofilo; Bifidobacterium bifidum ; B. longum; B. breve; B. infanti;	MIX PROBIOTICO
Irritable Bowel Syndrome and Prevents Gut	YangLiua	Cina	2020	Engineering	Probiotico	Lactobacillus plantarum	Lactobacillus plantarum
fermented milk with the probiotic	AlenkaŠmid	Slovenia	2016	2	Prebiotico	fermented milk	Prebiotico
containing Bifidobacterium	IrenaRoškar	Slovenia	2017	Journal of Functional Foods	Probiotico	plantarum MP2026	MIX PROBIOTICO
acidophilus in the treatment of irritable bowel	StéphaneSadrin	Francia	2020	Digestive and Liver Disease	Probiotico	Lactobacillus acidophilus	Lactobacillus acidophilus
cerevisiae versus placebo in the irritable	Chambrun	Francia	2015	Digestive and Liver Disease	Probiotico	Saccharomyces cerevisiae	Saccharomyces
bifidum MIMBb75 (SYN-HI-001) in the	ViolaAndresenMD	Germania	2020	Gastroenterology &	Probiotico	Bifidobacterium bifidum MIMBb75	Bifidobacterium
Monosaccharides and Polyols Improves	Shanti Eswaran	USA	2018	and Hepatology	Prebiotico	Foodmap	Prebiotico
diagnostic analyses moreeffective in reducing	Yuanyuan Wang	Cina	2018	in Medicine	Prebiotico	Erbal treatment	Prebiotico
Patients With Irritable Bowel Syndrome and A	Heidi MariaStaudacher	UK	2017	Gastroenterology	Simbiotico	Fodmap + mix probiotico	MIX SIMBIOTICO
are predictive of Low-FODMAP diet efficacy	AmeenEetemadi	USA	2021	Clinical Nutrition	Prebiotico	foodmap	Prebiotico
formulation of almond (Amygdalus dulcis L.)	SodaifDarvishmoghadam	Iran	2019	in Medicine	Prebiotico	Almond	Prebiotico
irritable bowel syndrome and the effect of low	Heidi M.Staudacher	UK	2019	Clinical Nutrition	Prebiotico	Foodmap	Prebiotico
Fig Effects on Irritable Bowel Syndrome with	Makan Pourmasoumi	Iran	2020	Explore (NY)	Prebiotico	FLIXWEED AND FIG	Prebiotico
Fecal Microbiome, and Markers of	Selina R.Cox	UK	2020	Gastroenterology	Prebiotico	Foodmap	Prebiotico
alleviates symptoms and reduces	Xu H	Cina	2020	Eur J Nutr	Probiotico	Bifdobacterium animalis subsp. lactis V9	MIX PROBIOTICO
viable tablet in the treatment of diarrhea	Bai T	Cina	2020	Trials	Probiotico	faecalise Bacillus cereus	MIX PROBIOTICO
Containing Lactobacillus and Bifidobacterium	Skrzydło-Radomańska B	Polonia	2020	Nutrients	Simbiotico	Lactobacillus and Bifidobacterium fodmap	MIX SIMBIOTICO
in IBS Patients	Goran Hauser	Croazia	2020	Clinical Trials	Probiotico	OMNi-BiOTiC STRESS	MIX PROBIOTICO
Irritable Bowel Syndrome	Shejal A Hanman	India	2020	Clinical Trials	Probiotico	paracasei DSM 24733, L. delbrueckii subsp. bulgaricus DSM 24734),	MIX PROBIOTICO
With Irritable Bowel Syndrome	Bożena Cukrowska	Polonia	2020	Clinical Trials	Probiotico	Bifidobacterium, Lactobacillus, Streptococcus species	MIX PROBIOTICO
Disorders in Childhood	Ruggiero Francavilla	Italia	2018	Clinical Trials	Probiotico	Lactobacillus Rhamnosus Strain GG	Lactobacillus rhamnosus
6475 in Moderate to Severe Irritable Bowel in	Pedro Gutierrez Castrellon	Spagna	2018	Clinical Trials	Probiotico	6475	Lactobacillus reuteri
intestinal bacterial overgrowth (SIBO)	AR Masjedizadeh	Iran	2018	Clinical Trials	Probiotico	E. coli strain Nissle	MIX PROBIOTICO
controlled trial of Saccharomyces cerevisiae	R Spiller	UK	2016	United European	Probiotico	Saccharomyces cerevisiae I-3856	Saccharomyces
reduces symptoms of irritable bowel	S Cui	Cina	2012	Clinical and Experimental	Probiotico	bifid triple viable capsule	MIX PROBIOTICO
faecal microbiota profiles in irritable bowel	C Stevenson	Sud africa	2016	Clinical Nutrition	Probiotico	Bacteroides spp., Bifidobacteria bifidum and Lactobacillus plantarum	MIX PROBIOTICO
pediatrics with recurrent abdominal pain	P Rahmani	Iran	2019	Gastroenterology	Probiotico	Lactobacillus reuteri	Lactobacillus reuteri
and microbiome in patients with irritable	Jin Young Yoon	Korea	2019	United European	Probiotico	Streptococcus thermophiles, Lactobacillus plantarum	MIX PROBIOTICO
microbiota composition of patients with	K Hod	Israele	2020	United European	Probiotico	Probiotic capsule (BIO- 25)	MIX PROBIOTICO
with daily journals for 12 weeks in a	N Ship	Canada	2019	Gastroenterology	Probiotico	Rhamnosus CLR2	MIX PROBIOTICO
on symptoms, gut microbiota, short chain	C Cremon	Italia	2014	United European	Probiotico	Lactobacillus paracasei CNCM I-1572	Lactobacillus paracasei
Children With Irritable Bowel Syndrome in a	Robert J. Shulman	USA	2019	Clinical Trials	Prebiotico	Psyllium	Prebiotico

Dietary Management of Irritable Bowel	Ece Mutlu, MD	USA	2019	Clinical Trials	Prebiotico	NTX-1 Fibe	Prebiotico
Pain-related Functional Gastrointestinal	Andrea Horvath	Polonia	2013	Clinical Trials	Prebiotico	GNN	Prebiotico
mixture (B-GOS®) on gastrointestinal	J Vulevic	UK	2018	Neurogastroenterology and	Prebiotico	B-GOS®	Prebiotico
barbadensis Mill. extract on symptoms, fecal	B Ahluwalia	Svezia	2020	Neurogastroenterology and	Prebiotico	Aloe Barbadensis	Prebiotico
hydrolyzed guar gum (PHGG) versus placebo	E Niv	Israele	2016	Clinical Trials	Prebiotico	PHGG	Prebiotico
